# Clinical Evaluation of Non-surgical Sterilization of Male Cats with Single Intra-testicular Injection of Calcium Chloride

**DOI:** 10.1186/1746-6148-7-39

**Published:** 2011-07-21

**Authors:** Kuladip Jana, Prabhat K Samanta

**Affiliations:** 1 Division of Molecular Medicine, Bose Institute, Centenary Campus, P 1/12, C.I.T. Scheme, VIIM, Kolkata-700 054, India; 2 Department of Surgery & Radiology, Faculty of Veterinary and Animal Sciences, West Bengal University of Animal & Fishery Sciences, 37 & 68, Kshudiram Bose Sarani, Kolkata- 700 037, India

**Keywords:** cat, calcium chloride, sterilization, androgenic key enzymes, stress, testosterone

## Abstract

**Background:**

Calcium chloride solution is an established injectable sterilant in dogs and other mammals. With cat populations a continuing problem, we sought to explore its first use in cats. Six cats per group were injected with 5%, 10% or 20% calcium chloride dihydrate in saline solution with lignocaine hydrochloride, a local anaesthetic.

**Results:**

At the 60^th ^day post-injection, cat testes were collected and showed complete testicular necrosis and replacement by fibrous tissue; very low sperm counts; and reduction of serum testosterone by at least 70% in 20% dose. Androgenic enzyme activities and their expressions were also reduced in all the treated groups along with intra-testicular testosterone concentration was also low. Increased testicular lipid peroxidation, with reduced antioxidants and mitochondrial membrane potential, were evident following calcium chloride treatments. However, there were no apparent changes in serum concentrations of cortisol, fasting blood sugar level, blood urea nitrogen, packed cell volume, or total serum protein following calcium chloride injection, suggesting that this method of sterilization is not associated with any general stress response.

**Conclusion:**

Calcium chloride solution demonstrates potential for androgenesis-eliminating nonsurgical sterilization of male cats in addition to its proven efficacy in dogs and other mammals.

## 1. Background

Sterilization has long been recognized as the most effective means of controlling pet populations. Yet with the huge numbers of owned and un-owned cats in the developing countries like India, the sterilization programs currently available are not enough. The mainstay of population control for male cats has been accomplished through surgical sterilization, namely orchidectomy (castration) [1]. However, for many reasons, surgical sterilization may not be effective as the sole method for population control. It requires anesthesia, medical equipment, a sterile surgical suite, a trained veterinarian, recovery time, incision site observation, and more [2]. It carries the risks that inherent in any surgical procedure. Furthermore, many people are unwilling to subject their pets to what they perceive to be a painful and invasive procedure. The cost of surgery is prohibitive for many owners, particularly in developing countries. In addition, when considering cat populations where permanent sterilization is desired, surgical methods can be expensive to be performed on a large scale [1]. Presently, a viable alternative to surgical sterilization is being intensively investigated. A non-surgical form of contraception is a promising additional method of pet population control. An ideal non-surgical sterilizing agent would be one which is safe, effective, affordable, permanent, and delivered in a single injection, with predictable effects on behavior and health [2,3].

In humans, an ideal chemo-sterilizing agent would be one which effectively arrests the production of sperm (spermatogenesis) or blocks their fertilizing capacity without inhibiting or altering the production of male steroid hormones (steroidogenesis), libido, accessory sex glands activities and pituitary function. But for other animals such as cats, an ideal chemical sterilizing agent would be one which arrest androgenesis and libido as well as spermatogenesis [4]. Otherwise the development of secondary sex characteristics may cause management problems of animals in the community, especially at their breeding season.

Sterilization by chemicals (chemical castration) is a non-surgical approach to male contraception. Chemical agents injected into the testis, epididymis or vas deferens cause infertility by inducing azoospermia in male animals. The technique is not technically demanding, is inexpensive and is suitable for large scale sterilizing programs in both domestic and wild animals [1].

Intra-testicular injection has been investigated as a method of inducing aspermatogenic orchitis and male contraception for more than five decades [1,5]. For example, injection of 10-25 units of freund's complete adjuvant (FCA) or Bacillus Calmette Guerin (BCG) directly into testis of male dogs resulted in severe oligospermia or azoospermia without the development of circulating anti-sperm antibodies. Infertility occurred within 6 weeks and lasted for several months [5,6]. In addition, intra-testicular administration of a combined solution of methallibure, dexamethasone, metopiron, niridazole, α-chlorohydrin or danazole caused testicular and epididymal atrophy and azoospermia in dogs [7]. Injection of 3.5% formalin in phosphate buffered saline or 1.5% chlorhexidine gluconate in 50% dimethyl sulfoxide into the tail of both epididymis in dogs resulted in irreversible azoospermia with chemical occlusion and secondary testicular atrophy [8]. Although intratesticular injection of glycerol resulted in sustained azospermia in squirrel monkey or Saimiri sciureus [4], even 70% glycerol solution did not result in azoospermia and sterility in dogs [9]. Intra-epididymal treatment with formalin alone induced only temporary azoospermia or oligospermia in treated dogs [8]. A single injection directly into the vas deferens with *sclerosing *chemical agents like 10% silver nitrate, 3.6% formaldehyde in ethanol, 5% potassium permaganate, 100% ethanol, or 3.6% formaldehyde resulted in irreversible infertility in male dogs [10] but no elimination of unwanted reproductive behavior. Intra-epididymal administration of zinc arginine also resulted in azoospermia within 90 days following injection in male dogs [11]. In 2003, the FDA approved a product Neutersol^®^, labeled for chemical castration via intra-testicular injection in male puppies. Neutersol^®^/Esterisol^®^, is a zinc-gluconate solution neutralized to a pH of 7 by arginine [1]. The procedure involves injecting a predetermined amount of zinc solution based on scrotal width into each testis of puppies 3-10 months of age [1]. Histological findings within 2.5 months of injection induced almost complete fibrosis of the seminiferous tubules and Leydig cells [1,12]. Though Neutersol^® ^is not currently available in the U.S., a similar compound, Esterisol^®^, is on the market in Mexico. However, neither compound produces a decline in testosterone long-lasting enough to significantly reduce nuisance behavior. Studies using these models of male contraception report no or minimal signs of discomfort following injection, but a transient increase in testicular diameter may follow the injection, resulting scrotum swelling. Additional local and systemic reactions reported after intra-testicular injections include scrotal ulceration and dermatitis, scrotal self-mutilation, preputial swelling, vomiting, diarrhea, anorexia, lethargy and leukocytosis [1]. Also, unlike surgical castration, this kind of chemical sterilization does not eliminate gonadal sources of testosterone [1]. In another study on Isabella Island of the Galapagos, 3.9% of 103 dogs given zinc gluconate developed necrotizing injection site reactions. By comparison, 3.4% of 58 dogs experienced wound dehiscence after surgical castration. The zinc gluconate reactions were more severe and required more extensive surgical repair than the traditional surgical complications. The dogs that experienced zinc gluconate reactions were large, mature dogs that received near maximum volume doses [13]. However, Neutersol^®^, the product is appealing to owners who do not want their dogs to have surgery or who want their dogs to retain the "masculine" look and presence of testicles.

Moreover, there are very few reports available in the literature related to the chemical sterilization of cats. Intra-epididymal injection of a 4.5% solution of chlorhexidine digluconate resulted in azoospermia or severe oligospermia in tom cats [14]. Unlike in the dog, transient scrotal swelling and pain persisted for up to 2 weeks following intra-epididymal injection in cats. Due to the above negative aspect following the use of the chemical, an effective chemosterilizing agent has yet to be established in cats. We thus began testing calcium chloride solution, reported in the U.S. veterinary literature as early as 1978 [15]. Based on reports and on its chemistry, we expected calcium chloride to be less toxic and carry less risk of skin necrosis and injection site reaction than previously-tested compounds, including zinc gluconate. We have reported that single bilateral intra-testicular injection of calcium chloride solution, with or without added local anesthetic, indeed induces permanent (irreversible) sterilization in male albino rats, goats and dogs through testicular degeneration, along with significant and dose-dependent diminution in testosterone concentration, without imposition of any apparent pain, general stress response, metabolic toxicity, or toxic and untoward side effects [2,3,16,17]. This simple technique thus fulfills the criteria of a method for nonsurgical sterilization in male animals. Previously, to study the mode of action of calcium chloride for induction of sterilization in other mammals we have seen that calcium chloride caused oxidative testicular tissue damage [2,3,16,17]. Nowadays, it is clear that the oxidative tissue damage occurred due to the increased production of reactive oxygen species (ROS) and ROS damages lipids, proteins, carbohydrates and DNA and also caused lipid peroxidation and affects enzyme activity and cellular genetic machinery [17]. Indeed, it has been shown that ROS inhibits steroidogenesis, steroidogenic enzyme activity and testosterone production in testis [16,17]. However, the biological systems possess a number of mechanisms to remove ROS, such as the integrated antioxidant defense systems. Different enzymatic and non-enzymatic scavengers of ROS, may protect the cellular system from the deleterious effects of the oxidative stress [16,17]. In continuation of our previous work, in this present study we investigate the effectiveness and also the mode of action of calcium chloride solution for chemical sterilization of male cats.

## Methods

### Animals

Clinically healthy 30 male cats (*Felis catus*) weighing 2-3 kg, aged from 09 to 12 months with normal libido in the breeding season (December-January) were acclimatized initially for one month in the animal house. Free roaming partially socialized cats were collected from the housing of animal lovers. The privately owned cats were temporarily donated for the study with known consent as per the guidelines of "Committee for the Purpose of Control and Supervision of Experiments on Animals (CPCSEA)", Government of India. Breeding soundness in all selected cats were judged and every cats showed sexual interest for the oestrus queen cat. The animals were routinely de-wormed with giving PRAZICON (Concept Pharmaceuticals, Mumbai, India; Paziquantel- 5 mg/kg body weight, Pyrantel pamoate- 15 mg/kg body weight, Febantel- 15 mg/kg body weight) and vaccinated with NOBIVAC^® ^Rabies (intervet, Hyderabad, India; Inactivated adjuvant vaccine against Rabies vaccination in cats) and NOBIVAC^® ^Tricat (intervet, Hyderabad, India; Combined live vaccine for cats against feline viral rhinotracheitis calcivirus infection and panleucopenia) prior to arrival in the animal housing area. The animal house has artificial lighting and controlled temperatures (22°C, ranging from 19 to 25°C and 12 h:12 h light dark cycles). The experimental protocol was approved by the ethical committee of the Faculty of Veterinary Surgery and Radiology, West Bengal University of Animal and Fishery Sciences. The cats were housed in pairs in indoor and outdoor runs, fed a standard commercial cat food and given water *ad libitum*. Investigations were conducted in accordance with the "Principles for the Care and Use of Research Animals" recommended by the Society for the Study of Reproduction. Guidelines for Ethical Conduct in the Care and Use of animals (American Psychological Association) were followed throughout the experimental duration. The animal house was registered with "Committee for the Purpose of Control and Supervision of Experiments on Animals (CPCSEA)", Government of India.

### Experimental protocol

**Figure 1:**
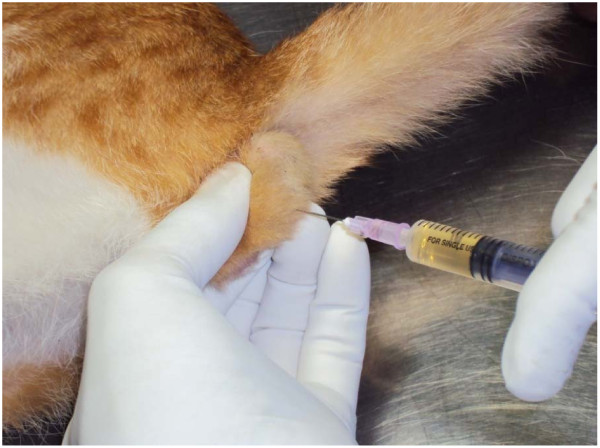
**Photograph shows the procedure of single bilateral intra-testicular injection of 0.25 ml sterile analytical grade of different concentrations of calcium chloride solution containing 1% lignocaine hydrochloride for sterilization of mature male cats**. Each intra-testicular injection was performed using a sterile 27-guage 1/2 in. needle that was directed from the ventral aspect of each testis approximately 0.5 cm from the epididymal tail towards the cranial aspect of that testis. The solution was carefully deposited along the entire route by linear infiltration while withdrawing the needle from proximal to distal end. Care was taken to prevent the seepage of the solution from the injection site.

The maximum effective dose of calcium chloride for induction of sterilization was estimated by dividing the 30 animals by random selection into 5 groups (Group I-V). Every animal in each of the 3 groups (Group I, II and III) received a single bilateral intratesticular injection of 0.25 ml of 5%, 10% or 20% sterile analytical grade of calcium chloride dihydrate in saline solution per testis (CaCl_2_, 2H_2_O, Merck, Mumbai, India) containing 1% lignocaine hydrochloride (a local anaesthetic agent, Astra IDL, Bangalore, India) respectively (Figure 1) under standard humane manual restraint condition guided by experienced Veterinary Surgeons. The injection volume of 0.25 ml was selected by standardization with a dose dependent study and in that volume of high dose calcium chloride solution caused necrosis of entire testicular gland parenchyma without any leakage from the gland. So that 0.25 ml of different concentration of calcium chloride were considered to evaluate the effectiveness of calcium chloride for the sterilization in cats. The animals in the control group (Group IV) each received a single bilateral intra-testicular injection of 0.25 ml sterile normal saline per testis containing 1% lignocaine hydrochloride. Parallel surgical castration was done in another group (Group-V) of animals according to the standard procedure.

### Intratesticular injection of calcium chloride solutions

Each intratesticular injection was performed using a sterile 27-guage 1/2 in. needle directed from the ventral aspect of each testis approximately 0.5 cm from the epididymal tail towards the cranial aspect of that testis. The solution was carefully deposited along the entire route by linear infiltration while withdrawing the needle from proximal to distal end. Necessary care should be taken to prevent the seepage of the solution from the injection site. In the previous study we have seen that if the solution was spilled in the scrotal skin then there was a skin lesion due to tissue necrosis. But if the solution immediately wiped away then the complications were avoided.

### Routine clinical observation

All the animals were kept under routine clinical observations for 60 days. We performed a routine clinical observation before and subsequent follow-up exams (Continuous observations for first 72 hours and after that with 1 hr. intervals up to 7 days, 6 hr intervals up to 30 days and of 12 hr intervals up to 60 days) after chemical and surgical sterilization procedure and must paid attention to the palpation of the testis. Following few parameters were sincerely considered during clinical observation.

1. Body weight 2. General attitude 3. Appetite 4. Rectal temperature 5. Scrotal and inguinal integument 6. palpation of testis and 7. Heart and Respiratory rate.

### Measurement of testicular volume

Length and width of the right and left testis were measured using laboratory callipers, and the volume of each testis was quantified by multiplying the length (cm) by width (cm^2^) by 0.524. Total testicular volume (cm^3^) was calculated by adding together the volume of the right and left testis [18].

### Collection of blood and testes

60 days following intra-testicular injection, both testes were obtained from all cats by castration under anaesthesia. Each animal was anesthetized with an intra-muscular injection of 20 mg/kg ketamine hydrochloride combined with 0.06 mg/kg diazepam. The both testicular weights were recorded and the gonado-somatic index was calculated with the ratio of testes weight and body weight. The right testis from each animal was used for histo-morphological studies, while the left one was used for biochemical assays. Blood was collected in 0, 30, 45 and 60 days after intratesticular injection before castration in fasted animals between 8.00 a.m. and 8.30 a.m. from the saphenous vein of each cat into a clean tube. A portion of blood was allowed to stand for 10-15 min at 4°C. The serum was aspirated into clean tubes and centrifuged 1500 × g for 10 min at 4°C. The serum samples were stored at -20°C until assayed for hormone concentrations.

### Epididymal sperm count

Sperms were collected from an equal length of the caudae of the excised epididymis of each cat by flushing through vas deferens with same volume (10 ml) of suspension medium containing 140 mmol NaCl, 0.3 mmol KCl, 0.8 mmol Na_2_HPO_4_, 0.2 mmol KH_2_PO_4 _and 1.5 mmol D-glucose (pH adjusted to 7.3 by adding 0.1 (N) NaOH) (E Merck). Collected sample was centrifuged at 100 × g for 2 min, and the precipitate part was re-suspended in 10 ml of fresh suspension medium. A fraction of suspension (100 μl) was mixed with an equal volume of 1% Trypan blue in the same medium, and numbers of sperms were counted in four chambers (used for counting of WBC) of the hemocytometer slide. At this concentration of Trypan blue (0.5%), the dye was completely excluded by intact sperms, which appeared bright and colourless, but taken up by dead and damaged sperms, which showed blue heads. The sperms number was expressed per ml of suspension [19].

### Histopathological studies on the testes

The right testis from each animal was fixed in Bouin's fixative and embedded in paraffin wax. A section 5 μm thick was cut from the middle portion of each testis, stained with hematoxylin-eosin and examined under light microscopy at 100X and 400X magnifications. The structures of the seminiferous tubules and interstitial spaces in the testis were examined.

### Assays for serum and intratesticular testosterone and cortisol concentrations

The testicular tissue was homogenized in 0.5 ml of water using Teflon homogenizer that was fitted into a microfuge tube chilled in ice. Each sample was centrifuged at 10000 rpm for 10 min. The supernatant was removed, frozen, and stored until the hormone assay [20]. The serum and intratesticular concentrations of testosterone and cortisol were measured using an ELISA reader (Merck, Japan) according to the standard protocol given by *National Institute of Health and Family Welfare *(NIHFW, New Delhi, India) [21]. The ELISA kit for testosterone was supplied by IBL (Hamburg, Germany) and the cortisol kit was supplied by NIHFW. Horseradish peroxidase was used as an enzyme-labelled antigen in both assays that made a competition with unlabelled antigen for binding with a limited number of antibody sites on the micro-plates (solid phase). Assays were performed following standardized instructions. Each testosterone concentration was calculated from a standard curve with 5 standards supplied by IBL whereas each cortisol concentration was calculated from 6 standards supplied by NIHFW with the absorbance of the standards and samples monitored against a blank at 450 nm. The stated cross-reaction of the testosterone antibody with dehydrotestosterone was 10% and the intra-assay CV was 6.2%. The stated cross-reaction of the cortisol antibody with corticosterone was 10% and intra-assay CV was 5.5%. All the samples were included in a single assay.

### Estimation of total serum protein concentrations, blood urea nitrogen and fasting blood sugar levels

Total serum protein concentration was measured according to the standard method of Lowry *et al. *[22] and the level was expressed as gm/dl. Blood urea nitrogen was measured using the kit supplied by Dr. Reddy's Laboratories (diagnostic division), Hyderabad, India, according to the manufacturer's instructions. Fasting blood sugar level was measured using a single touch glucometer (Blood Life Scan, Johnson and Johnson, Milpitas, California, USA) and the concentration was expressed as mg/100 ml.

### Steroidogenic enzymes activities

3β-Hydroxysteroid dehydrogenase (3β-HSD) and 17β-hydroxysteroid dehydrogenase (17β-HSD) are the two crucial enzymes in the steroid biosynthesis pathway and they are the key androgenic enzymes in the testis. These two enzymes were assayed according to the methods described earlier [19]. One unit of the enzyme activity was equivalent to a change in the absorbance of 0.001 units/min at 340 nm.

### Western Blot Analysis of testicular steroidogenic enzymes

The testicular tissue was homogenized in approximately 20 volumes of buffer using a tissue protein extraction kit (Pierce Chemical Co. Rockford, IL, USA) and cleared by centrifugation at 10000 × g for 10 min at 4°C to produce a protein extract. The protein content of the preparation was determined with a BCA kit (Pierce) using BSA as a standard. An equal concentration of each sample (100 μg protein) was resolved on 10% sodium dodecyl sulfate- poly-acrylamide gels (SDS- PAGE) and electrophoretically transferred to nitrocellulose membranes. The membrane was blocked for 1 hr. in TBST (0.05% Tween-20 in Tris-buffered saline, pH 7.6) with 5% non-fat milk and then incubated with 1: 100 specific primary antibodies (rabbit polyclonal 3β-HSD and 17β-HSD antiserums, Santa Cruz Biotechnology Inc., Santa Cruz, CA, USA) at 4°C overnight and subsequently exposed to horseradish peroxidase- conjugated anti rabbit IgG goat poly-clonal secondary antibody. In between each step, the membrane was washed with TBST about 10 minutes for three times. The bands were visualized with the Amersham Pharmacia Biotech enhanced chemiluminescence system (Uppsala, Sweden) according to the manufacturer's instructions. Finally the developed blots were subjected to densitometry using the β-actin (Specific β- actin antibody, Cell Signalling Technology, Beverly, MA, USA) as an internal control [23].

### Assay of testicular glutathione peroxidase (GPx) activity

The activity of glutathione peroxidase was determined by the slightly modified method of Rotruck *et al. *[24]. Briefly, the assay mixture containing 0.5 ml of sodium phosphate buffer, 0.1 ml of 10 mM sodium azide, 0.2 ml of 4 mM reduced glutathione, 0.1 ml of 2.5 mM H_2_O_2_, and 0.5 ml 1:10 tissue extract was taken and the total volume was made up to 2 ml with distilled water. The tubes were incubated at 37°C for 3 min and the reaction was terminated by the addition of 0.5 ml 10% TCA. To determine the residual glutathione content, the supernatant was removed after centrifugation and to this, 4.0 ml of disodium hydrogen phosphate (0.3 M) solution and 1 ml of DTNB reagent were added. The colour developed was measured at 412 nm in a spectrophotometer against a blank containing only phosphate solution and DTNB reagents. The enzyme activity was expressed as units/mg of protein (1 unit is the amount of enzyme that converts 1 μmol GSH to GSSG in the presence of hydrogen peroxide/min).

### Assay of testicular glutathione reductase (GR) activity

The activity of glutathione reductase was determined using the method of Staal *et al. *[25]. Briefly, the assay mixture containing 0.2 ml of tissue extract, 1.5 ml of sodium phosphate buffer, 0.5 ml of 25 mM EDTA, 0.2 ml of 12.5 mM oxidized glutathione, and 0.1 ml of 3 mM NADPH were prepared, and immediately read at 340 nm in a spectrophotometer against a blank containing all the components except the enzyme at 3 min at 30 s interval. The activity of GR was expressed as μmol of NADPH oxidized/min/mg of protein.

### Assay of testicular glutathione-S-transferase (GST) activity

The enzymatic activity was determined with the method of Habig *et al. *[26]. Briefly, the assay mixture containing 0.4 ml of potassium phosphate buffer, 0.1 ml of tissue extract, 1.2 ml of distilled water and 0.1 ml of 1-chloro-2, 4-dinitrobenzene (CDNB) was added and incubated in a water bath at 37°C for 10 min. After incubation, 0.1 ml of 30 mM reduced glutathione was added. Immediately, the optical density was measured against a blank at 340 nm on a spectrophotometer at 30 s interval for 3 min. The activity of GST was expressed as units/mg of protein (1 unit is the amount of enzyme that conjugate 1 nmol of CDNB with GSH/min).

### Assay of testicular glucose-6-phosphate dehydrogenase (G-6-PDH) activity

The enzyme was assayed by the method of Beutler [27]. Briefly, the assay mixture containing 0.1 ml each of Tris-HCl buffer, NADP and MgCl2, 0.5 ml of water and 0.1 ml of 1:5 diluted tissue extract was taken in a cuvette. The reaction was started by the addition 0.1 ml of glucose-6-phosphate and the increase in optical density was measured at 340 nm against a blank. The activity of G-6-PDH was expressed as μmol of glucose-6-phosphate to 6-phosphogluconate/min/mg protein.

### Assay of testicular γ- glutamyl transpeptidase (γ- GT) activity

The testicular tissue was homogenized in ice-cold 0.1 mol Tris Hcl buffer, pH 7.4 at a tissue concentration of 10%/ml and then the homogenized mixture was centrifuged at 10,000 × g for 30 min at 4°C. The supernatant was used for the enzyme estimation. The enzyme activity determined by the modified method of Orlowski and Meister [28] and was expressed as μmol of *p*-nitroaniline formed/min/mg protein.

### Determination of lipid peroxidation

The level of lipid peroxidation was measured by the slightly modified method of Devasagayam and Tarachand [29]. In brief, the reaction mixture consisted of 1.0 ml of 0.15 M Tris-HCl buffer (pH 7.4), 0.3 ml of 10 mM KH_2_PO_4_, and 0.2 ml cell extract in a total volume of 2 ml. The tubes were incubated at 37°C for 20 min with constant shaking. The reaction was stopped by the addition of 1 ml 10% trichloroacetic acid. The tubes were shaken well, followed by addition of 1.5 ml thiobarbituric acid (TBA) and were heated in a boiling water bath for 20 min. The standard tubes containing 10, 20, 30, 40, and 50 nmol/ml were also run simultaneously. The tubes were centrifuged and the colour developed was measured at 532 nm. The malondialdehyde (MDA) content of the sample was expressed as nmoles of MDA formed per milligram protein.

### Quantification of testicular contents of reduced glutathione (GSH) and glutathione disulphide (GSSG)

GSH (reduced glutathione) and GSSG (oxidized glutathione) levels of testes were estimated as described by Hissin and Hilf [30]. To 0.5 ml of the tissue extract (10000 **× ***g *supernatant), 4.5 ml of the phosphate-EDTA buffer, pH 8.0, was added. The final assay mixture (2.0 ml) contained 100 μl of the diluted tissue supernatant, 1.8 ml of phosphate-EDTA buffer, and 100 μl of the 0.1% orthopthalaldehyde (OPT) solution. For GSSG estimation, 0.5 ml portion of the tissue extract was incubated at room temperature with 200 μl of 0.04 M *N-*ethyl maleimide (NEM) for 30 min to interact with GSH present in the tissue. To this mixture, 4.3 ml of 0.1 N NaOH was added. 100 μl of this mixture was taken and added to 1.8 ml of 0.1 N NaOH and 100 μl of the 0.1% OPT solution. After thorough mixing and incubation at room temperature for 15 min, the solution was transferred to a quartz cuvette. The fluorescence at 420 nm was determined with the activation at 350 nm. The tissue GSH and GSSG levels were obtained from a standard curve prepared using GSH and GSSG standards. The results were expressed as GSH/GSSG ratio.

### Determination of mitochondrial membrane potential (Δψ*m*)

Mitochondrial membrane potential (Δψ_m_) was measured using JC-1 probe according to the method described by Mishra and Shaha [31]. JC-1 is a vital cationic mitochondrial dye that is lipophilic and becomes concentrated in the mitochondria in proportion to the membrane potential; more dye accumulates in mitochondria with greater Δψ_m _and ATP generating capacity. Therefore, the fluorescence of JC-1 can be considered as an indicator of relative mitochondrial energy state. The dye exists as a monomer at low concentrations (emission, 530 nm, and green fluorescence) but at higher concentrations forms J-aggregates (emission, 590 nm, red fluorescence). The 530:590 ratio in fluorescence spectrophotometer (Perkin-Elmer) was considered as the relative Δψ_m _value.

### Statistical Analysis

The results were expressed as mean ± standard error (S.E.). One-way analysis of variance (ANOVA) with Bonferroni modification was first carried out to test for any differences between the mean values of all groups. If any differences between groups were established, the values of the treated groups were compared with those of the control group by a multiple two tail *t*-test. A value of *p *< 0.05 was interpreted as statistically significant [32].

## Results

All the cats did not suffer from fever or any noticeable complications except for a slight increase of firmness of a testis on palpation. Most of the cats showed signs of mild discomfort approximately 1 to 5 min after initiating calcium chloride as well as normal saline injections. Probably it may be caused by fluid pressure. Mild testicular swelling was evident in every cat by 24 h following injection. Swelling was maximum in treated animals up to 48 to 72 h following injection, then gradually decreased at 4 weeks (Data not presented). Every one of the cats injected with calcium chloride survived in good health throughout the experimental period.

### Changes in Gonado-somatic index (Testes mass and body weight ratio)

**Figure 2:**
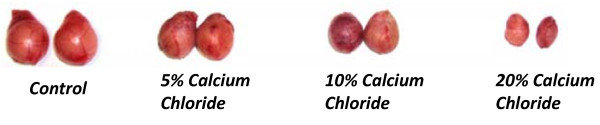
**Photograph showing comparative state of the testicular volume following 60 days of single bilateral intra-testicular injection of 0.25 ml normal saline, or 5%, 10%, or 20% sterile analytical grade of calcium chloride solution containing 1% lignocaine hydrochloride in mature male cats**. All the animals were kept for 60 days under routine clinical observations. Both the testes were collected from all cats by castration under anaesthesia.

A single intratesticular injection of calcium chloride at doses of 5, 10 or 20% led to a graded significant diminution (p < 0.01) in gonado-somatic index in comparison to the animals treated with only normal saline (vehicle control). This parameter exhibited a greater level of diminution at the 10 and 20% dose in respect to 5% dose treatments (Table 1). Furthermore, significant dose-dependent decrease was also observed in gross testicular volume following calcium chloride injection with respect to normal saline injection. A very small testicular mass was surgically removed from the 20% calcium chloride treated animals in comparison to the other dose treatments (Figure 2)

**Table 1:** Effects on gonado-somatic index, packed cell volume (PCV), total serum protein concentration, fasting blood sugar level, serum cortisol and epididymal sperm count following 60 days after single bilateral intratesticular injection of calcium chloride and surgical castration in male cats

Condition	Gonado-somatic index (Testes mass and body weight ratio; %)	Packed cell volume (PCV) (%)	Total serum protein (gm/dl)	Fasting blood sugar level (mg/dl)	Blood urea nitrogen (mg/dl)	Serum Cortisol (μg/ml)	Epididymal sperm count (No./ml)
Control	0.081+ 0.006	25.56 + 1.12	6.78 + 0.42	62.45 + 2.11	14.05 + 1.43	4.35 + 0.92	6540 + 580
5% Calcium Chloride	0.068 + 0.005*	25.86 + 1.32	6.85 + 0.31	62.73 + 1.78	13.69 + 0.98	4.72 + 0.81	4250 + 218*
10% Calcium Chloride	0.045 +0.004**	26.10 + 1.61	7.10 + 0.45	61.95 + 2.14	14.56 + 1.52	4.84 + 0.76	1124 + 42**
20% Calcium Chloride	0.026 + 0.006**	26.18 + 1.84	7.26 + 0.54	62.21 + 1.62	14.42 + 1.31	4.86 + 0.95	102 + 15**
Surgical Castration	****	26.05+ 2.11	6.73 + 0.65	61.87 + 1.56	14.24 + 1.74	4.90 + 1.12	****

Data presented as Mean + SE, n = 6.

(* p < 0.05, ** p < 0.01, as compared with their respective control).

### Changes in histomorphology of testis

**Figure 3:**
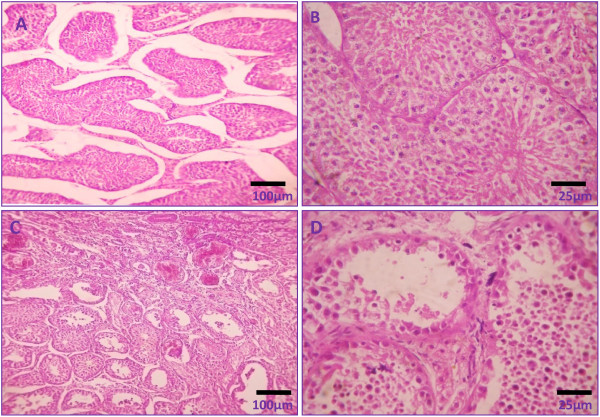
**Photomicrographs showing testicular histology following 60 days after single bilateral intra-testicular injection of 0.25 ml normal saline containing 1% lignocaine hydrochloride in mature male cats showed normal arrangement of germ cells in seminiferous tubules with distinct interstitial spaces (A & B) or 5% sterile analytical grade of calcium chloride solution containing 1% lignocaine hydrochloride in mature male cats induced derangement of germ cell association in seminiferous tubules along with washing out of germ cells from the tubules (C & D)**.

**Figure 4:**
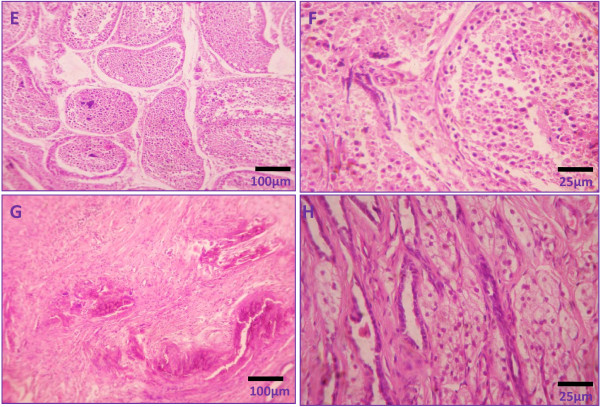
**Representative photomicrographs showing testicular histology following 60 days after single bilateral intra-testicular injection of 0.25 ml 10% sterile analytical grade of calcium chloride solution containing 1% lignocaine hydrochloride in mature male cats showed coagulative necrosis in the seminiferous epithelium and the interstitial spaces as well as presence of degenerated and coagulated germ cells in combination with fibrous tissue and infiltration of leucocytes in tubular and interstitial spaces (E & F) or 20% sterile analytical grade of calcium chloride solution containing 1% lignocaine hydrochloride in mature male cats resulted complete testicular necrosis along with only presence of fibrous tissue and hyaline tissue and neither presence of germ cells or Leydig cells (G & H)**.

Histology of testicular sections demonstrated normal stages of spermatogenesis in vehicle control groups with distinct interstitial spaces (Figure 3(A) & (B)). However, calcium chloride treatment induced severe degenerative changes in testicular tissue both in seminiferous tubules and interstitial cells of Leydig. The intratesticular injection of 5% calcium chloride induced dissolution of the germ cell association with atrophy in seminiferous tubules and washing out of the germ cells from the tubules. Some of the tubules showed the elimination of all germ cells and the presence of only spermatogonia and Sertoli cells. Yet the induced damage was uneven and tubules affected inconsistently in the lowest dose (Figure 3(C) &2(D)). Histomorphological analysis of testes of cats treated with 10% calcium chloride showed significant histomorphological changes, including coagulative necrosis in the seminiferous epithelium and the interstitial spaces as well as presence of degenerated and coagulated germ cells in combination with fibrous tissue in tubular and interstitial spaces (Figure 4(E & F)). The degeneration of seminiferous tubular architecture along with infiltration of leucocytes (mostly neutrophils) throughout the testicular tissue was also noted (Figure 4(E & F)). Intratesticular injection of 20% calcium chloride solution resulted in complete testicular necrosis of whole germinal epithelium with only presence of fibrous tissue and hyaline tissue. Moreover, the de-arrangement of tubular architecture without any distinct boundary between the tubular and extra tubular compartments was also noted. There was no identification of mature or immature germ cells in testicular sections. Besides this, there was no sign of regeneration in germ cells and interstitial Leydig cells (Figure 4(G & H)).

### Effect on epididymal sperm count

The epididymal sperm count was decreased significantly (p < 0.01) in all the calcium chloride treated cats in comparison to vehicle control animals. Diminution in the numbers of epididymal sperm after 10 and 20% of calcium chloride treatment was more drastic (p < 0.001) in respect to other dose treatment (Table 1).

### Effect on serum concentrations of testosterone and cortisol

**Figure 5:**
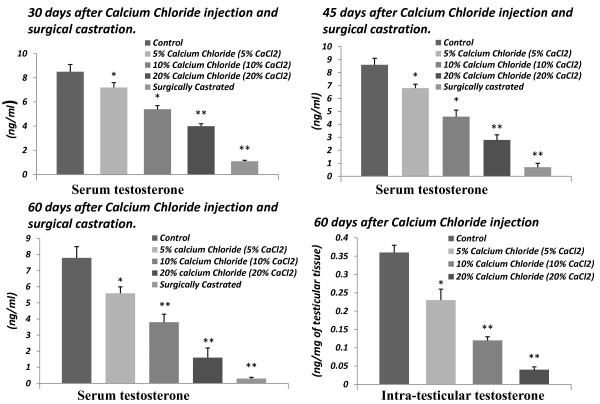
**Diagrammatic representations of the changes in serum testosterone (ng/ml) and intra-testicular testosterone concentrations (ng/mg of testicular tissue) following surgical castration as well as single bilateral intra-testicular injection of 0.25 ml 5% or 10% or 20% sterile analytical grade of calcium chloride solution containing 1% lignocaine hydrochloride in different time points (after 30 or 45 or 60 days) in mature male cats**.

Single intra-testicular injection of calcium chloride in cats resulted in decrease in serum and intra-testicular testosterone concentrations in a dose and duration dependent manner. A significant (p < 0.01) diminution in serum concentrations of testosterone was noted in the entire calcium chloride treated group as well as castrated group in comparison to control. Moreover, the 10 and 20% of calcium chloride treatments also exhibited a more effective and significant inhibition on these parameters in comparison to 5% calcium chloride treatment. The remarkable low level of serum and intra-testicular concentrations of testosterone was noted in 60 days after the highest dose of calcium chloride treatment (Figure 5). There was no significant change in serum concentration of cortisol at any of the doses of calcium chloride as well as castrated group in comparison to vehicle control as well as among the treated and castrated groups (p > 0.05; Table- 1).

### Effect on fasting blood sugar, blood urea nitrogen, PCV and total serum protein concentrations

There were no alterations in fasting blood sugar, blood urea nitrogen, PCV or total serum protein concentration in any of the treated groups and castrated group with respect to the control group as well as among the four treated and castrated groups (p > 0.05; Table 1).

### Effect on testicular 3β- HSD and 17 β - HSD expressions and activities

**Figure 6:**
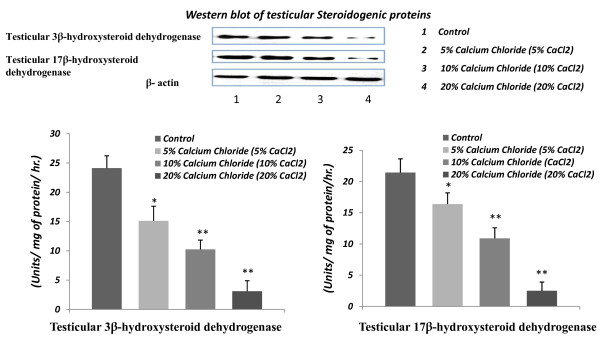
**Western blot analysis of testicular androgenic enzymes (3β-HSD and 17β-HSD) expressions and diagrammatic representations of the changes in these enzymes activities (units/mg of protein/hr.) following 60 days after single bilateral intra-testicular injection of 0.25 ml normal saline or sterile analytical grade of different concentrations of calcium chloride solution containing 1% lignocaine hydrochloride in mature male cats for induction of sterilization**.

Graded inhibitory responses from calcium chloride treatments were noted in testicular 3β- HSD and 17 β - HSD expressions and activities. The inhibitory responses from calcium chloride injection in the expressions of 3β- HSD and 17 β - HSD were noted through western blot analysis of testicular proteins. Moreover, the activities of 3β- HSD and 17 β - HSD in testicular tissue were decreased significantly (p < 0.01) in all the calcium chloride treated groups in respect to vehicle control. Besides this, the 20% of calcium chloride treatment also exhibited a more effective and significant inhibition on these parameters in comparison to 5% or 10% of calcium chloride treatments (Figure 6).

### Effect on testicular glutathione peroxidase (GPx), glutathione reductase (GRd), glutathione S-transferase (GST), glucose-6- phosphate dehydrogenase (G-6-PDH) and γ- glutamyl transpeptidase (γ- GT) activities

**Figure 7:**
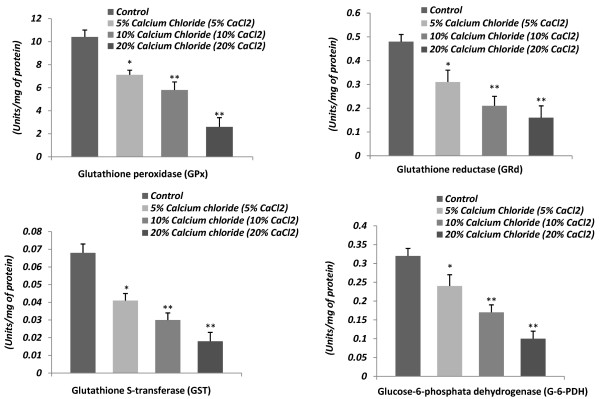
**Representative histograms showing the changes in testicular activities of glutathione peroxidase (GPx; units/mg of protein), glutathione reductase (GRd; units/mg of protein), glutathione S-transferase (GST; units/mg of protein), glucose-6-phosphate dehydrogenase (G-6-PDH; units/mg of protein) following 60 days after single bilateral intra-testicular injection of 0.25 ml normal saline or sterile analytical grade of different concentrations of calcium chloride solution containing 1% lignocaine hydrochloride in mature male cats for sterilization**.

**Figure 8:**
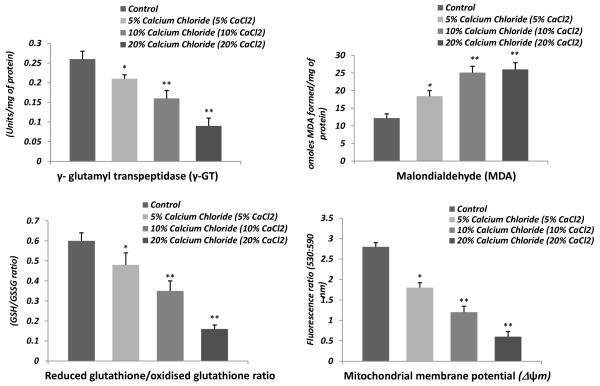
**Representative histograms showing the changes in testicular γ- glutamyl transpeptidase (γ-GT; units/mg of protein) activity, MDA levels (nmoles of MDA fomed/mg of protein), GSH/GSSG ratio (Reduced glutathione/oxidised glutathione ratio) and mitochondrial membrane potential (Δψm; fluorescence ratio at 530:590 nm) following 60 days after single bilateral intra-testicular injection of 0.25 ml normal saline or sterile analytical grade of different concentrations of calcium chloride solution containing 1% lignocaine hydrochloride in mature male cats for sterilization**.

A dose-related drastic inhibitory response on the testicular antioxidant status was noted following calcium chloride injections. The testicular activities of GPx, GRd, GST, G-6-PDH and γ- GT were significantly (p < 0.001) decreased in calcium chloride treated cats in comparison with the controls, indicating the suppressed testicular antioxidant defense against ROS, which facilitated the induction of oxidative stress. The dose of 20% caused a greater degree of inhibition in the activity of these enzymes. Significant inhibition (p < 0.01) in these testicular enzyme activities was noted for every dose from 5 to 20% calcium chloride in comparison to vehicle control (Figure 7 &8).

### Changes in testicular content of malondialdehyde (MDA)

Testicular content of MDA was elevated (p < 0.01) after treatment with 5% calcium chloride in respect to the controls, but the 10 or 20% dose exerted the greatest degree of elevation (p < 0.001) in the testicular content of MDA when compared to the controls. However, levels of MDA in testicular tissue were not significantly different between 10% and 20% of calcium chloride treatments (Figure 8).

### Changes in testicular content of reduced glutathione (GSH) and oxidized glutathione ratio and mitochondrial membrane potential (Δψm)

The testicular content of GSH and GSSG ratio and mitochondrial membrane potential (Δψ*m*) were decreased significantly (p < 0.01) in response to calcium chloride treatment in each of the doses with respect to the controls (Figure 8). Moreover, drastic diminutions in these parameters were noted in the 20% dose of calcium chloride treatments.

## Discussion

**Figure 9:**
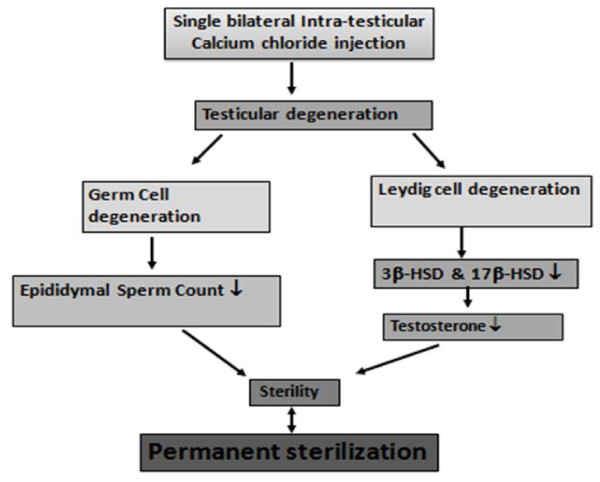
**Diagrammatic representation of the mechanism related to induction of sterilization of male cats following single bilateral intra-testicular injection of calcium chloride**.

Although intra-testicular injections have been investigated as a method of contraception in pet animals for more than five decades, this study is the first to show a potent androgen-eliminating sterilization method in cats and its mode of action (Figure 9). When considering cat populations where permanent sterilization is desired, surgical castration can be expensive to be performed on a large scale. Moreover, this is the only study published so far reporting the sterilization of male cats by single bilateral intratesticular injection of calcium chloride. The maximum responses in biochemical and histological parameters related to sterilization were noted at the 20% dose when compared with surgical castration. Calcium chloride induces coagulative necrosis of the entire testicular tissue. This is in agreement with previous studies with this chemical agent on the testes in the rat [3,16] and in other domestic animals [2,17]. Both the calcium chloride and vehicle-treated animals showed signs of mild discomfort up to 5 approximately min following injection due to the excessive fluid pressure. The sings of discomfort were temporary. Contrary to what a layperson might assume, afferent nerve endings associated with pain sensation are located only on the scrotal skin and in the capsule of the testis rather than within the testicular and epididymal parenchyma. This anatomical arrangement would make a pain response from within the test is unlikely [33]. However, these nerve endings may have been stimulated as intra-testicular pressure increased during and immediately following injection. Moreover, to subside this problem 1% lignocaine hydrochloride, a local anesthetic is used in the injection.

Testicular histology also showed the degenerative changes associated with the graded doses of calcium chloride. The disintegration of germ cell association in seminiferous tubules and washing out of germ cells from the seminiferous tubules were noted even with the lowest dose, though the tubular compartment remained distinct with respect to the extra-tubular compartment. Drastic necrosis in seminiferous tubules along with atrophy of the tubules was noted starting with the 10% dose. But after the high dose (20%) of calcium chloride treatment, complete degeneration of germ cells was observed, together with the absence of a distinct boundary of seminiferous tubules with respect to the interstitial spaces along with appearance of fibrous tissue and hyaline tissue. These changes may be due to the necrotizing properties of calcium chloride as reported by others [34-36]. In addition, germ cell degeneration by calcium chloride has been associated with low serum concentrations of testosterone, a prime regulator for the maintenance of structural morphology as well as the normal physiology of seminiferous tubules [37-39]. The induction of testicular degeneration by this chemical agent is supported by the diminution in gonado-somatic index in treated cats, when this is an accepted measure of testicular damage [2,17]. The graded and significant diminutions in the serum concentrations of testosterone in response to graded doses of calcium chloride were correlated with graded diminutions in the expressions and activities of testicular 3β- HSD and 17β- HSD, especially as these are key enzymes for testicular androgenesis [19]. The degeneration in the interstitial cells of Leydig by the graded doses of calcium chloride may have led to a reduction in serum testosterone concentrations [40]. The low concentrations of serum testosterone in cats treated with calcium chloride was further evidenced by the qualitative study of testicular sections in which significant fibrosis was seen with the medium and highest doses. These have been due to a low concentration of testosterone [41]. Moreover, the low serum concentration of testosterone in calcium chloride treated cats has been further indicated here by significant diminution in epididymal sperm count, as sperm maturation in the epididymis is controlled by testosterone [38]. The efficacy of calcium chloride in inducing sterilization was supported by the necrosis of the seminiferous tubules and interstitial cells, along with the significant fibrosis and hyalinization. These effects are consistent with previous studies using other chemical agents for sterilization [9,42]. Infiltration of leucocytes into the seminiferous tubules and interstitial spaces after calcium chloride treatment may have been due to damage of the testicular tissue or to degeneration that may have released large amounts of chemotactic factors responsible for the ingression of leucocytes [43].

Another factor in the sterilization induced by calcium chloride could be the generation of large amounts of free radicals, or their products, in the testicular tissue. Free radical production in the testis results in a low level of testosterone [44,45]. Calcium chloride is also an important chemical agent for inducing the generation of free radicals in tissues [46]. Free radicals can cause the destruction of all cellular structures and of lipids by lipid peroxidation [47]. The extent of lipid peroxidation and consequently the associated tissue damage can be assessed by the measurement of MDA levels [48,49]. The fact that an intratesticular injection of calcium chloride was associated with free radical production and lipid peroxidation in the testis was reflected by the high testicular content of MDA. The alternative way by which free radicals may be generated in the testis would be due to the infiltration of leucocytes. Testicular free radical production has been closely associated with the infiltration of leucocytes [50,51]. This elevation in the formation of free radicals in testes treated with calcium chloride is also supported by the diminution in testicular GPx, GRd, GST, G-6-PDH and γ-GT activities, as these are considered important scavenging enzymes against free radicals in male gonads [23,51,52]. Besides these enzymatic antioxidants, calcium chloride treatment was also associated with a lowered content of testicular reduced glutathione (GSH), as important non-enzymatic antioxidant in testis [53]. The low activities of testicular GPx, GRd, GST, G-6-PDH and γ-GT along with a low content of GSH among the treated cats may have been be due to the lowered concentrations of testosterone [44,45]. It may be assumed that testicular degeneration in this experiment was due to generation of free radicals in the testes, as free radicals are inhibitors for spermatogenesis [23,54] and testicular androgenesis [16]. In addition, the reduced GSH/GSSG ratio of testes would be another cause for the degenerative necrosis of seminiferous tubules. Glutathione is important regulators for proliferation and differentiation of germ cells and provide protection of germ cells against harmful effects of free radicals [55,56]. Degeneration of these cells by calcium chloride treatment could be a possible cause of the lower content of glutathione in the treated testis. The observed decreases in the levels of enzymatic and non-enzymatic antioxidants are consistent with our past studies [2,3,16,17]. In the present study, the decreased level of GSH induced by calcium chloride can seriously impair the optimal functioning of these various catalytic activities of GSH dependent enzymes [23]. Moreover, the elevated level of ROS can cause oxidation of the mitochondrial pore and thereby disrupt the mitochondrial membrane potential (Δψm) [31]. These unique observations emphasize that calcium chloride can have turned on one particular initiating mechanism to execute all the effects observed, and the mechanism appears to be calcium chloride-induced testicular degeneration. The elevated MDA and the decline in the levels of antioxidants are consistent with this conception [23].

Stress indicators including the concentrations of serum cortisol, total serum protein, blood urea nitrogen, fasting blood sugar and PCV were measured to ascertain whether the calcium chloride treatment was associated with any general stress response in the experimental animals [57]. As there were no significant alterations in the serum concentrations of cortisol, blood urea nitrogen, fasting blood sugar level, PCV or total serum protein concentrations in the animals treated with calcium chloride or surgically castrated with respect to the controls, this method of sterilization does not appear to be associated with any general stress response, which is also supported by our previous findings [2,3,16,17]. Until now gonadectomy has been the only option for permanent sterilization of cats all over the world, but this is the first report of an androgen-eliminating nonsurgical method of sterilization of male cats. Due to its permanent reduction of testosterone, calcium chloride would be used in cases where elimination or reduction of sex-based behavior is desired, such as for family pets and in control cat programs. The primary practical advantages of calcium chloride over surgery are affordability, elimination of the need for anesthesia, sterile conditions, and recovery care. It is easy to inject since unlike previous attempts at sterilizing agents, it is not rapidly caustic to the skin; little training is required and complications are avoided if any spilled solution is wiped away. The primary disadvantage is slow onset of action (4-6 weeks) and the level of discomfort during injection, which we hope to address by calibrating injection volume more specifically in near future.

## Conclusion

A single bilateral intra-testicular injection of calcium chloride solution is effective, economical, easy to perform and does not require to removal of testis in cats. It causes permanent sterilization and is a simple alternative method to surgical castration.

## Authors' contributions

KJ and PKS both have made substantial contributions equally to conception and design, or acquisition of data, or analysis and interpretation of data and preparation of this manuscript. All authors read and approved the final manuscript.
